# Early onset torsion dystonia (Oppenheim's dystonia)

**DOI:** 10.1186/1750-1172-1-48

**Published:** 2006-11-27

**Authors:** Christoph Kamm

**Affiliations:** 1 Department of Neurodegenerative Diseases, Hertie-Institute for Clinical Brain Research, University of Tübingen, Hoppe-Seyler Str. 3, 72076 Tübingen, Germany

## Abstract

Early onset torsion dystonia (EOTD) is a rare movement disorder characterized by involuntary, repetitive, sustained muscle contractions or postures involving one or more sites of the body. A US study estimated the prevalence at approximately 1 in 30,000. The estimated prevalence in the general population of Europe seems to be lower, ranging from 1 in 330,000 to 1 in 200,000, although precise numbers are currently not available. The estimated prevalence in the Ashkenazi Jewish population is approximately five to ten times higher, due to a founder mutation. Symptoms of EOTD typically develop first in an arm or leg in middle to late childhood and progress in approximately 30% of patients to other body regions (generalized dystonia) within about five years. Distribution and severity of symptoms vary widely between affected individuals. The majority of cases from various ethnic groups are caused by an autosomal dominantly inherited deletion of 3 bp (GAG) in the *DYT1 *gene on chromosome 9q34. This gene encodes a protein named torsinA, which is presumed to act as a chaperone protein associated with the endoplasmic reticulum and the nuclear envelope. It may interact with the dopamine transporter and participate in intracellular trafficking, although its precise function within the cell remains to be determined. Molecular genetic diagnostic and genetic counseling is recommended for individuals with age of onset below 26 years, and may also be considered in those with onset after 26 years having a relative with typical early onset dystonia. Treatment options include botulinum toxin injections for focal symptoms, pharmacological therapy such as anticholinergics (most commonly trihexiphenydil) for generalized dystonia and surgical approaches such as deep brain stimulation of the internal globus pallidus or intrathecal baclofen application in severe cases. All patients have normal cognitive function, and despite a high rate of generalization of dystonia, 75% of those patients are able to maintain ambulation and independence, and therefore a comparatively good quality of life, with modern treatment modalities.

## Disease name and synonyms

Early onset torsion dystonia

Oppenheim's dystonia

Dystonia musculorum deformans

## Definition

Early onset torsion dystonia (EOTD) is characterized by involuntary, repetitive, sustained muscle contractions or postures, typically of the limbs, which may spread to other body parts, in the absence of other neurological abnormalities. It is inherited in an autosomal dominant fashion with a penetrance of only 30–40% [1], suggesting that various genetic and/or environmental factors contribute to development of the disease. The majority of cases are caused by a deletion of 3 bp (GAG) in the *DYT1 *gene on chromosome 9q34.

The term "Dystonia" in general refers to a large diverse group of movement disorders involving involuntary muscle contractions or postures. It may be classified in three ways:

(1) clinically, *i.e. *according to age at onset or distribution of affected body regions;

(2) etiologically, *i.e. *into primary, secondary, dystonia-plus or heredodegenerative;

(3) genetically, with 15 loci described to date.

## Diagnostic criteria

The following diagnostic criteria have been established for EOTD [1-3]:

### Definite dystonia

Characteristic overt twisting or directional movements and postures that are consistently present.

### Probable dystonia

Postures or movements suggestive of dystonia that are insufficient in intensity or consistency to merit classification as definite (*e.g. *excessively tense and labored writing with minimal posturing, flurries of blinking but no episodes of sustained closure, *etc.*).

### Possible dystonia

Muscle contractions not considered abnormal but remotely suggestive of dystonia (*e.g. *unusual hand grip with mild excess hand tension but normal flowing handwriting, increased blinking with no flurries or sustained contractions, *etc.*).

Only the category of "definite dystonia" was shown to be 100% specific in *DYT1 *families [3], suggesting that only those patients with definite signs of dystonia should be considered affected in genetic research studies.

## Epidemiology

EOTD is a rare disease, with an estimated prevalence of 3.4:100,000 and annual incidence of 0.2:100,000 in Rochester, Minnesota [4]. The prevalence in the Ashkenazi Jewish (AJ) population is estimated to be five to ten times higher [5], due to a founder mutation which appears to have arisen approximately 350 years ago in Byelorussia/Lithuania [6]. The frequency of the *DYT1 *GAG deletion in the AJ population is high, between 1:2000 and 1:6000. In contrast, the prevalence in the general population of Europe seems to be lower. Estimates range from 0.3 to 0.5:100,000 although precise numbers are currently not available.

## Clinical description

EOTD is characterized by involuntary, repetitive, sustained muscle contractions or postures involving one or more sites of the body. Typically, symptoms develop first in an arm or leg in middle to late childhood (mean age of onset: 12.5 years) [7] and progress in approximately 30% of patients to other body regions (generalized dystonia) within about five years [8]. "Torsion" refers to the twisting nature of body movements observed in EOTD, often affecting the trunk. Patients may be severely physically incapacitated, but are normal intellectually and show no other neurological symptoms. Distribution and severity of symptoms vary widely between affected individuals, ranging from mild focal dystonia, *e.g. *writer's cramp [9] to severe generalized dystonia, even within families [10]. Earlier age of onset and onset in the legs predicts a more severe clinical course, *i.e. *development of generalized dystonia [11,12].

The *DYT1 *GAG deletion is responsible for the majority of typical early onset dystonia cases of diverse ethnic origins, whether inherited or caused by a *de novo *mutation, whereas only a small minority of patients with atypical, *e.g. *focal dystonia, harbor this mutation [7,13-18]. If mutant gene carriers pass the age of approximately 28 years without developing symptoms, they usually escape the disease for life.

## Etiology

Neuropathologically, EOTD is characterized by a lack of neurodegeneration, suggesting that neuronal dysfunction, rather than loss of neurons, underlies disease symptoms. However, cell bodies of dopaminergic neurons appear to be enlarged in brains from dystonia patients [19]. In the brains of four *DYT1 *patients, perinuclear inclusion bodies in specific brainstem areas (pedunculopontine nucleus, periaqueductal gray) have been reported [20]. These findings have been recapitulated in two different *DYT1 *animal models, both in transgenic mice overexpressing human mutant ΔE-torsinA [21] and in male *DYT1 *ΔGAG knock-in mice [22]. Despite the low penetrance of only 30–40% for clinical symptoms, functional imaging studies found metabolic hyperactivity in the lentiform nuclei, cerebellum and supplemental motor areas, and impaired sequence learning in both manifesting and non-manifesting carriers of the *DYT1 *GAG deletion, indicating that penetrance of the GAG deletion is greater than that assumed based on clinical grounds [23,24]. In addition, a recent study found that striatal D_2 _receptor binding was reduced in non-manifesting carriers of the *DYT1 *GAG deletion. This reduction may be a trait feature of *DYT1 *dystonia, with other factors being necessary for manifestation of the signs and symptoms. Alternatively, clinical penetrance may be linked to the extent of the decrease in striatal D_2 _receptor binding [25].

The *DYT1 *gene encodes the torsinA protein. TorsinA is an endoplasmic reticulum/nuclear envelope (NE) ATP-dependent chaperone protein [26,27] involved in intracellular trafficking [28]. Mutant ΔE-torsinA relocalizes to the NE [27,29,30], and postmigratory neurons from both torsinA null and homozygous mutant knock-in mice develop membrane abnormalities of the NE [31]. In addition, recent studies in mammalian cell lines have shown that overexpression of wild-type torsinA appears to regulate the intracellular trafficking of the dopamine transporter, whereas overexpression of mutant ΔEtorsinA had no effect. These results establish a potential link between torsinA and the dopaminergic system [59].

Several lines of evidence suggest that the disease is caused by a dysfunction of the basal ganglia: (1) Although torsinA is ubiquitously expressed, expression levels within the human brain are highest within dopaminergic neurons of the substantia nigra [32]; (2) Striatial [^18^F]dopa uptake is mildly reduced in manifesting *DYT1 *GAG deletion carriers [33]; (3) An increase in the ratio of dopamine metabolites to dopamine was found in the postmortem striatum of *DYT1 *dystonia brains as compared to controls [34]; (4) A phenotypically similar form of inherited dystonia, dopa-responsive dystonia (DRD), is caused by insufficient synthesis of dopamine [35,36], and symptomatic dystonia frequently affects patients with acquired lesions of the basal ganglia (*e.g. *stroke) [37,38].

## Diagnostic methods

Molecular genetic diagnostic in conjunction with genetic counseling is recommended for individuals with age of onset below 26 years, and may also be considered in those with onset after 26 years having a relative with typical early onset dystonia [7].

## Differential diagnosis

Differential diagnosis includes other forms of primary dystonia and disorders in which dystonia is one of several neurological conditions present. The second large and diverse group includes the dystonia-plus syndromes, the heredodegenerative dystonias, the secondary ("symptomatic") dystonias, other dystonia-like conditions, and early-onset parkinsonism.

### Other forms of primary dystonia (DYT2, 4, 6, 7 and 13)

The phenotype of ***DYT2 ***dystonia, an autosomal recessive form of dystonia, resembles early onset torsion dystonia (*DYT1*) in three of the four families reported to date (early onset of symptoms, frequently in the feet, followed by rapid generalization) [39,40]. However, there is a debate as to whether the mode of inheritance in these families is actually autosomal recessive or autosomal dominant with reduced penetrance [41].

The ***DYT4 ***locus has been assigned to an Australian family with predominant whispering dysphonia and additional variable dystonic symptoms [42,43], clearly different phenotypically from classical *DYT1 *dystonia.

***DYT6 ***refers to another autosomal dominant form of dystonia of mixed-type described in two German-Mennonite families [44]. In contrast to *DYT1 *dystonia, the average age at onset in these families was higher (18.6 years, range 5–38), and in many patients dystonic symptoms were predominantly cranio-cervical, with progression to generalized dystonia in only a few cases.

***DYT7 ***refers to a locus for adult-onset focal, predominantly cervical dystonia mapped in a German family [45].

The ***DYT13 ***locus has been mapped in a non-Jewish Italian family [46] with mostly segmental dystonia with prominent cranio-cervical and arm involvement, more closely resembling the *DYT6 *than the *DYT1 *phenotype.

### Dystonia-plus (DYT5, DYT11 and DYT12)

Dystonia-plus refers to conditions in which dystonia is one of only two neurological abnormalities present, the other usually being either parkinsonism or myoclonus. This group includes Dopa-Responsive Dystonia (DRD, ***DYT5***), Myoclonus-Dystonia (***DYT11***) and Rapid Onset Dystonia-Parkinsonism (***DYT12***).

### Heredodegenerative dystonias (DYT3 and others)

In heredodegenerative dystonias, dystonia is part of a more widespread neurodegenerative syndrome, often with known inheritance, and may or may not be a prominent feature. This large group includes X-linked Dystonia-Parkinsonism ("Lubag", *DYT3*), Idiopathic Parkinson's Disease (IPD), Multiple System Atrophy (MSA), Progressive Supranuclear Palsy (PSP), Hallervorden-Spatz Disease, Wilson's disease, Rett syndrome, Neuroacanthocytosis, and many others.

### Secondary dystonia

Secondary (symptomatic) dystonias are caused by environmental insults such as stroke, tumors, infections, drugs and toxins or by metabolic disorders (*e.g. *homocysteinuria, metachromatic leukodystrophy, Lesch-Nyhan syndrome, and others).

### Other dystonia-like conditions (DYT8, DYT9, and DYT10)

This group includes conditions mimicking symptoms of dystonia (*e.g. *psychogenic dystonia or pseudodystonia) and paroxysmal disorders, such as paroxysmal non-kinesigenic dystonia (PNKD, *DYT8*), paroxysmal choreoathetosis with episodic ataxia and spasticity (*DYT9*), paroxysmal kinesigenic dystonia (PDK, *DYT10*), and others.

### Early onset parkinsonism

A considerable proportion of early onset Parkinson's disease, with onset of symptoms before 40 years of age, is caused by mutations in the *Parkin *gene [47] and frequently presents with dystonia, especially in the lower limbs [48]. Thus, this condition should be considered in the differential diagnosis of EOTD.

## Genetic counseling

Based on a systematic genetic and clinical analysis of 267 individuals with primary torsion dystonia, including Ashkenazi Jewish and non-Jewish patients, diagnostic *DYT1 *testing in conjunction with genetic counseling is recommended for individuals with an age of onset below 26 years, and may also be considered in those with onset after 26 years having a relative with typical EOTD [7]. Genetic counseling should take into account that *DYT1 *dystonia is inherited in an autosomal dominant manner with a low penetrance of 30–40% and great clinical variability. Few cases of *de novo *mutations have been reported [49,50]. Asymptomatic adult relatives should be informed before testing that onset of *DYT1 *dystonia after age 26 is very unlikely, and that, if it occurs, symptoms are usually mild and unlikely to progress [7]. Testing of asymptomatic at-risk individuals during childhood is not recommended. In addition to prenatal testing through chorionic villus sampling, preimplantation genetic diagnosis has recently been reported [51].

## Management including treatment

### Drug therapy

As DRD (*DYT5*) is phenotypically very similar to EOTD, but its symptoms, in contrast to EOTD, typically respond very well to dopaminergic therapy, it is recommended that every patient with early onset focal dystonia (onset below 26 years) be given a trial of L-Dopa/decarboxylase inhibitor, starting with 1 mg/kg per day of levodopa and slowly increasing until complete benefit is achieved or dose-limiting side effects appear. Most DRD patients respond well to 4–5 mg/kg per day [52].

In general, drug therapy in EOTD is not very effective. Anticholinergics, especially in a high dosage (*e.g. *trihexiphenydil up to 30–60 mg/day), are the only substance class for which controlled studies are available [53]. Dosage should be increased very slowly to prevent side effects such as cognitive or memory deficits, which are frequently dose-limiting, although children can generally tolerate much higher doses than adults. Other substances, which may or may not be effective, include baclofen, benzodiazepines such as diazepam or clonazepam, pimozid or tetrabenazin. In severe cases of segmental or generalized dystonia, especially of the lower body and trunk, intrathecal baclofen administration may be considered [54].

### Botulinum toxin injections

Independent of genetic background, local botulinum toxin injections directly into affected muscles now represent the first line treatment of focal dystonias (reviewed in [55]). Therefore, botulinum toxin injections by trained movement disorder specialists may be a useful therapy for selected muscle groups in EOTD, such as neck muscles for torticollis, facial muscles for blepharospasm or hand/arm muscles for writer's cramp. If successful, the injections need to be repeated on a regular basis (usually every 3–6 months).

### Surgical approaches

Due to technical advances in recent years, interest in functional surgical approaches in dystonia has been renewed, in particular deep brain stimulation (DBS) of the globus pallidus and pallidotomy (reviewed in [56]). Although experience with this approach is still limited, preliminary results in patients with primary generalized dystonia, especially *DYT1 *GAG deletion carriers, are very promising [57]. Therefore, DBS may be helpful in selected EOTD patients with severe generalized dystonia.

## Prognosis

Only few studies on the natural history of EOTD in genetically confirmed patients have been conducted. In one report following the clinical course of 33 patients from Israel [58], 63.6% had progressed into generalized dystonia after a mean of 15.5 +/- 13.8 years of symptoms, 15% were wheelchair-bound and 9% were using walking aids. All patients had normal cognitive function. Despite the high rate of generalization, 75% of those patients were able to maintain ambulation and independence, and therefore a comparatively good quality of life, with modern treatments combining drugs, botulinum toxin and functional neurosurgery.

## Unresolved questions

Very little is known yet about the normal cellular function of the torsinA protein. Further studies are required to address how mutant torsinA causes neuronal dysfunction, and how this mutation leads to a dominantly inherited disease with a markedly low penetrance and a very selective neurological phenotype. Since the age of onset is associated with a period of motor learning and high synaptic plasticity, a possible role of torsinA in coordinated neuronal development merits further investigation. In addition, further evidence for or against direct involvement of the dopaminergic system in EOTD is warranted.
